# *mLife*: Your journal for cutting‐edge research in all microbiological disciplines

**DOI:** 10.1002/mlf2.12005

**Published:** 2022-03-16

**Authors:** Li Huang, Jizhong Zhou

**Affiliations:** ^1^ State Key Laboratory of Microbial Resources, Institute of Microbiology, Chinese Academy of Sciences Beijing China; ^2^ College of Life Science, University of Chinese Academy of Sciences Beijing China; ^3^ Institute for Environmental Genomics, University of Oklahoma Norman OK USA; ^4^ Department of Microbiology and Plant Biology, University of Oklahoma Norman OK USA; ^5^ School of Civil Engineering and Environmental Sciences, University of Oklahoma Norman OK USA; ^6^ Earth and Environmental Sciences, Lawrence Berkeley National Laboratory Berkeley CA USA

After years of deliberations, the Institute of Microbiology of the Chinese Academy of Sciences (IMCAS; http://www.im.cas.cn) and the Chinese Society for Microbiology (CSM; http://www.csm1952.org.cn/) decided early in 2021 to jointly launch *mLife*, a new high‐profile open‐access microbiology journal with a scope spanning the entire spectrum of microbiological sciences. An editorial board consisting of over 70 internationally well‐established scientists with expertise in various areas of microbiology has been established. It is a great honor for both of us to work with our outstanding board members in getting this new journal off the ground.

Microbes, including bacteria, archaea, fungi, protists and viruses, are the most diverse group of life on Earth, inhabiting almost every imaginable environment. They are vital to Earth ecosystems, and affect every aspect of our life. The study of microbes (i.e., microbiology), as a whole, has experienced ups and downs over the past century. In the last two decades, the rapid development and wide application of high‐throughput sequencing, various omics, genome editing techniques, imaging, single‐cell and single‐molecule technologies, and so forth have revolutionized the analysis of microbes across different scales, that is, from molecules and cells to populations, communities, ecosystems, and biosphere. Consequently, we know much more now than 20 years ago about microbes with respect to their biochemistry, genetics, physiology, diversity, ecology, and evolution. It has also been increasingly recognized that our understanding and exploitation of these creatures are critical to the development of bioeconomy, fighting emerging and re‐emerging infectious diseases, such as the ongoing Covid‐19 pandemic, and maintaining ecosystem functioning and service. Clearly, microbiology contributes significantly to the health of both humankind and Earth.

Active research in microbiology has been reflected in a substantial increase in the number of microbiology publications in recent years. In fact, the expansion of microbiology literature is among the fastest in life sciences over the past 5 years. In keeping pace with high publication demand, the number of microbiology journals has also increased. However, integrative journals for the publication of topnotch research across all microbiology disciplines are still lacking. At the suggestion of the editorial board, *mLife* will set out to publish the best quality and most significant research in all microbiological disciplines as well as in interdisciplinary fields involving microorganisms. The Journal welcomes manuscripts reporting first‐class basic and applied research on microbial life. It also encourages submissions concerning new fields in microbiology (e.g., microbiomics) and interdisciplinary fields (e.g., synthetic biology, geomicrobiology, use of big data and artificial intelligence in microbiology, etc.). Since the progress of microbiology depends increasingly on technological and methodological innovations, a special category of “Methods and Instrumentation” is also set up. Efforts will be made to ensure the adequate representation of different fields in the journal.

Somewhat surprisingly, *mLife* is the first English microbiology journal with a scope covering all disciplines in microbiology from China, which has been ranked among the top nations in the world that lead in the number of microbiology publications over the last 5 years. The launch of this Journal has been enthusiastically supported by the Chinese microbiology community. Although the use of microorganisms in fermentation practices in China, such as wine making and soy sauce brewing, dates back to thousands of years ago, modern microbiology was introduced into the country only at the beginning of the last century. However, the past four decades have witnessed a rapid growth in microbiology research in China, thanks in part to international cooperation and scientific exchanges. Both IMCAS and CSM are esteemed microbiological organizations in China. IMCAS, founded in 1958, is the largest microbiological institution in China, and CSM, founded in 1952, has a total of ∼20,000 members at present. We are confident that, with strong support from these two organizations, *mLife* will have a smooth sail.

*mLife* will strive for excellence in microbiology publication, aiming to become among the best journals in the field of microbiology. *mLife* will adhere to the highest standards of scientific integrity and publication ethics. It will provide authors with the best professional service available in the publication industry, including rapid review, figure polishing, language improvement, and rapid and wide dissemination. We look forward to seeing the best science from you in *mLife*, which is your journal for cutting‐edge research in all microbiological disciplines.

